# Long-term effectiveness and moderators of a web-based tailored intervention for cancer survivors on social and emotional functioning, depression, and fatigue: randomized controlled trial

**DOI:** 10.1007/s11764-017-0625-0

**Published:** 2017-07-11

**Authors:** Roy A. Willems, Ilse Mesters, Lilian Lechner, Iris M. Kanera, Catherine A. W. Bolman

**Affiliations:** 1 Faculty of Psychology and Educational Sciences, Open University of the Netherlands, P.O. Box 2960, 6401 DL Heerlen, The Netherlands; 2 CAPHRI Care and Public Health Research Institute, Maastricht University, P. O. Box 616, 6200 MD Maastricht, The Netherlands

**Keywords:** Psycho-oncology, Survivorship, RCT, eHealth, Computer tailoring, Self-management

## Abstract

**Purpose:**

The web-based computer-tailored *Kanker Nazorg Wijzer* (Cancer Aftercare Guide) supports cancer survivors with psychosocial issues during cancer recovery. The current study investigates whether the 6-month effects in increasing emotional and social functioning and reducing depression and fatigue hold at 12 months from baseline. Moreover, it explores whether patient characteristics moderate the 6- and 12-month intervention effectiveness.

**Methods:**

Cancer survivors from 21 Dutch hospitals (November 2013–June 2014) were randomized to an intervention (*n* = 231) or a wait-list control group (*n* = 231). Intervention effects on emotional and social functioning (EORTC QLQ-C30), depression (HADS), and fatigue (CIS) were evaluated through multilevel linear regression analyses.

**Results:**

At 12 months from baseline, the intervention group no longer differed from the control group in emotional and social functioning, depression, and fatigue. Moderator analyses indicated that, at 6 months, the intervention was effective in improving social functioning for men (*d* = 0.34), reducing fatigue for participants ≤56 years (*d* = 0.44), and reducing depression for participants who received chemotherapy (*d* = 0.36). At 12 months, participants with a medium educational level reported higher social functioning (*d* = 0.19), while participants with a low educational level reported lower social functioning (*d* = 0.22) than participants with a similar educational level in the control group.

**Conclusions:**

The intervention gave cancer patients a head start to psychological recovery after the end of cancer treatment. The control group caught up in the long run.

**Implications for cancer survivors:**

The Cancer Aftercare Guide expedited recovery after cancer treatment. Being a low intensity, easy accessible, and relatively low cost intervention, it could serve as a relevant step in recovery and stepped care.

**Electronic supplementary material:**

The online version of this article (doi:10.1007/s11764-017-0625-0) contains supplementary material, which is available to authorized users.

## Introduction

After treatment, many cancer survivors face a variety of difficulties and challenges affecting their quality of life [1, 2], of which anxiety, depression, and fatigue are prominent issues [3–6]. Ideally, cancer survivors should have an active role in managing their health and well-being [7]. However, they often feel neither confident [8] nor prepared by health professionals to effectively manage life after cancer treatment, resulting in prominent information needs [9]. Since the number of cancer survivors will only increase over the years [10, 11], effective support in self-management is crucial.

The Internet has become an important source of health management information for cancer survivors [12, 13]. Survivors indicate being positive about self-management eHealth interventions [14]. A great advantage of such interventions is their potentially wide reach, easy accessibility, 24/7 availability, and anonymity [15]. In addition, personalized information on reaching the desired health outcome can be provided by means of computer tailoring [15], facilitating behavior change and/or maintenance [16]. eHealth interventions can be valuable to serve as a relevant step in stepped oncology care, since, in general, they are low in intensity and sufficient to meet the needs of a large proportion of survivors with relatively mild complaints. They can also easily be used for some patients to become aware of their need for more intensive interventions (e.g., therapist treatment or medication) [17, 18].

There is little evidence on the benefits of eHealth interventions for cancer survivors [19]. A few studies evaluated the effectiveness of stand-alone (i.e., not combined with face-to-face support) web-based psychosocial interventions [20–27], often only reporting preliminary results [20–24]. Three sufficiently powered randomized controlled trials found eHealth interventions for cancer survivors to be effective in reducing psychological distress [26, 27] and fatigue [26, 27], and improving quality of life [27] and self-efficacy skills [25]. Only one of these three interventions was developed for multiple cancer types and provided tailored information [27]. Carpenter et al. [25] and van den Berg et al. [26] found effects directly after intervention completion (10 weeks and 4 months after baseline, respectively), but not at any follow-up measures (20 weeks [25] and 6 and 10 months [26] after baseline). The follow-up effects for the intervention group of the study by Carpenter et al. [25] are, however, difficult to interpret, since the control group received access to the intervention before the last measurement. Yun et al. [27] also found effects directly after intervention completion (12 weeks after baseline), but they did not conduct any follow-up measures. More research on the long-term effectiveness of web-based interventions for cancer survivors is therefore needed [19].

Since effective self-management interventions for cancer survivors are highly needed, we developed the web-based computer-tailored *Kanker Nazorg Wijzer* (KNW; Cancer Aftercare Guide). The KNW provides cancer survivors personalized information and support on psychosocial and lifestyle issues [28–30]. Through problem identification, goal selection, psycho-education, action planning, and evaluation, survivors are encouraged to effectively manage life after cancer. The effectiveness of the KNW on quality of life, anxiety, depression, and fatigue was assessed 6 months after baseline in a randomized controlled trial (RCT) comparing an intervention group to a wait-list control group [29]. Participants had access for 6 months and were free to use the KNW whenever they wanted. In practice, 84% only used the KNW modules in the first 18 weeks following first login. The KNW was found to be effective in improving the quality of life components emotional and social functioning, and reducing depression and fatigue.

For future implementation of the KNW and evaluation of the impact of the intervention, further insight is needed into the extent to which the effects are sustained in the longer term. The main purpose of the current study is therefore to evaluate whether the effects on emotional and social functioning, depression, and fatigue at 6 months after baseline are sustained in the long term (i.e., 12 months after baseline). Since there is little evidence on the long-term effectiveness of web-based self-management interventions for cancer survivors, the current study contributes to the knowledge on this area. Second, intervention effectiveness might differ among patient subgroups [31]. While there is some evidence that demographic and treatment-related characteristics may influence effectiveness of psycho-oncological interventions [31, 32], there is, to our knowledge, no specific evidence whether these factors influence the effectiveness of web-based self-management interventions for cancer survivors specifically. This information is important, since this provides directions for further development of the tailored content used within the KNW. If the KNW is only effective for particular subgroups, then providing additional subgroup specific information might improve intervention effectiveness. In the current study, we explore whether gender, age, educational level, and treatment type moderated intervention effectiveness at the short term (i.e., 6 months after baseline), as well as the long term (i.e., 12 months after baseline).

## Methods

The long-term effects were evaluated in an RCT comparing an intervention group with a waiting list control group. The RCT was registered in the Dutch Trial Register (NTR3375) and approved by the Medical Ethics Committee Zuyderland-Zuyd (NL41445.096.12).

### Intervention

The KNW (http://www.kankernazorgwijzer.nl) was systematically developed using the Intervention Mapping protocol [33]. This protocol consists of six steps: needs assessment, specification of objectives, selecting theories and applications, producing materials, program implementation, and evaluation. The KNW was developed as a stand-alone web-based intervention that aims to increase cancer survivors’ quality of life by providing psychosocial support and promoting positive lifestyle changes. The intervention consists of eight modules, of which seven are self-management training modules. The training modules cover the topics returning to work, fatigue, anxiety and depression, social relationship and intimacy issues, physical activity, diet, and smoking cessation. The eighth module provides general information on the most common residual symptoms. For an overview of the scope and sequence of all modules, see Online Resource 1. A detailed description of the study protocol and intervention components is published elsewhere [28].

The KNW is fully automated and computer tailored. Prior to using the program, participants fill in a baseline questionnaire that enables tailoring. Participants then receive personalized advice on which of the modules deserve their attention [34]. Within a module, the refinement of information is continued, eventually resulting in a personalized action plan. Further, the KNW is programmed to be an open and unrestrictive program: users can choose which modules they want to visit or which assignments they want to make.

The structure and the content of the training modules are based on the principles of problem solving therapy (PST) [35] and cognitive behavioral therapy (CBT) [36]. For PST, the modules consist of four components, divided over two sessions. In the first session, participants (1) identify their problem, (2) select a goal and receive psychoeducation and assignments on how to deal with their problem, and (3) personalize their goal through action plans. (4) After 30 days, participants are invited for a second session in which they can evaluate the progress of their goal. Basic CBT principles are covered by providing psycho-education, several assignments (e.g., monitoring behavior or thoughts, challenging dysfunctional cognitions, planning pleasant activities, setting new goals), and relaxation exercises. CBT-based assignments are mainly implemented in modules discussing issues with large psychosocial and cognitive components (i.e., return to work, fatigue, anxiety and depression, and social relationships and intimacy issues). The information provided in the modules is supported by videos of fellow survivors and professionals from different fields discussing recovery after cancer and dealing with problems and daily troubles.

### Participants and procedure

Patients were eligible for participation if they were 18 years or older; they had been diagnosed with any type of cancer; their primary treatment (surgery, chemotherapy, and/or radiotherapy) had been completed successfully for at least 4 weeks but no more than 56 weeks; there was no sign of recurrence in the latest follow-up visit; they were able to speak and read Dutch; there was no serious medical, psychiatric, or cognitive illness that would interfere participation; and they returned a signed informed consent form.

Representatives of 45 hospitals in the Netherlands (e.g., department heads, oncologists, research nurses, nurse practitioners) from outpatient clinics internal medicine, oncology, gynecology, urology, and the breast clinic were contacted for assistance in recruitment. Professionals of 21 hospitals recruited patients between November 2013 and June 2014. The professionals had access to patient files and often knew the patients personally and thus were able to determine whether a particular patient met the inclusion criteria and was physically or mentally able to participate in the study. Eligible patients were invited to participate by giving them an information package during a follow-up visit or sending the package following review of the patient’s files. The information package included (1) a letter with trial information and a username and password for first login, (2) an informed consent form with return envelope, (3) an information brochure concerning Medical Research, (4) an instruction manual on how to use the KNW, and (5) a card with contact details. A reminder was sent after 2 weeks. Patients who agreed to participate were requested to return the signed consent form to the Open University of the Netherlands. Sample size calculations were based on the outcomes quality of life, anxiety, and depression and showed that, after correction for multilevel analyses and an expected dropout of 20%, 188 patients per group were required (*α* = .10, *β* = .20, *d* = 0.30).

After online registration, the computer randomly assigned participants to either the intervention or the waiting list control group (allocation ratio 1:1). Participants were not stratified before group assignment. Both groups had to fill in a questionnaire at baseline and after 3, 6, and 12 months from baseline. The 3-month measurement measured possible mediating variables [37], while the 6- and 12-month measurement aimed to measure the short- and long-term effectiveness of the intervention, respectively. The intervention group had access to the KNW for 6 months directly after baseline. Access to the intervention was postponed for the waiting list control group until after the 12-month measurement.

### Measurements

*Demographic characteristics* included gender, age, relationship status, educational level, income level, and employment status. Educational level was categorized as “low” (lower vocational education, medium general secondary education), “medium” (secondary vocational education, higher general secondary education) and “high” (higher vocational education, university education), according to the Dutch educational system. *Disease-related characteristics* included body mass index (BMI), cancer type, having had cancer before, treatment type, time since last treatment, participation in support program after treatment, and comorbidity. As the majority of participants had breast cancer, cancer type was dichotomized into “breast” and “other” (i.e., bladder, colorectal, esophageal, gynecologic, hematologic, kidney, liver, lung, prostate, stomach, testicular, and thyroid cancer). Treatment type was categorized as “surgery and chemotherapy,” “surgery and radiotherapy,” “surgery, chemotherapy, and radiotherapy,” and “other” (see Table 1).

**Table 1:** Baseline demographic and disease-related characteristics (*n* = 462)

	Control (*n* = 231)	Intervention (*n* = 231)
Demographic characteristics		
Gender (% women)	80.5%	79.2%
Age (years) (mean ± *SD*)	56.16 ± 11.33	55.59 ± 11.46
Relationship status (% partner)	79.7%	83.5%
Education level (%)		
Low	42.0%	32.9%
Medium	30.3%	32.9%
High	27.7%	34.2%
Modal income (%)		
Below modal income	18.2%	12.1%
Approximately modal income	33.8%	36.4%
Above modal income	48.1%	51.5%
Employment status (% employed)	48.1%	52.8%
Disease-related characteristics		
BMI (mean ± *SD*)	26.45 ± 4.86	25.96 ± 4.96
Cancer type		
Breast	71.0%	70.1%
Bladder	0.4%	0.4%
Colorectal	15.6%	12.6%
Esophageal	1.3%	1.3%
Gynecologic	2.6%	3.9%
Hematologic	6.1%	5.2%
Kidney	0.4%	1.3%
Liver	0.4%	0%
Lung	0%	2.2%
Prostate	0.9%	1.3%
Stomach	0.9%	0.4%
Testicular	0.4%	0.9%
Thyroid	0%	0.4%
Had cancer before (% yes)	10.0%	10.4%
Treatment type		
Surgery	13.4%	12.1%
Chemotherapy	4.3%	3.5%
Radiotherapy	1.3%	0.4%
Chemotherapy and radiotherapy	0.4%	0.4%
Surgery and chemotherapy	20.8%	26.4%
Surgery and radiotherapy	12.3%	19.9%
Surgery, chemotherapy, and radiotherapy	46.8%	37.2%
Time since last treatment (weeks) (mean ± *SD*)	23.44 ± 12.90	25.06 ± 13.49
Participation in support program (% yes)	61.0%	62.8%
Comorbid condition (% yes)	27.3%	26.8%

*Emotional and social functioning* were measured with the EORTC Quality of Life Questionnaire (EORTC QLQ-C30) [38]. The emotional functioning scale (four items, *α* = .88) assessed whether participants felt tense, irritable, depressed, or were worried. The social functioning scale (two items, *α* = .77) assessed whether the participants’ physical condition or treatment had interfered with their family life or social activities. Items in both scales were measured on a 4-point scale. Total scale scores ranged from 0 to 100. A high score represents a high level of functioning.

*Depression* was measured with the Hospital Anxiety and Depression Scale (HADS) [39]. Items (seven items, *α* = .82) were measured on a 4-point scale. Scale score ranged from 0 to 21, with a score of 8 or higher being an indication for depression.

*Fatigue* was measured with the total score of the Checklist Individual Strength (CIS) [40]. The 20-item CIS comprises four scales measuring subjective fatigue, concentration, motivation, and activity. All items range from 1 to 7. The total score (range 20–140, *α* = .94) is an overall indication of fatigue, with a score of 77 or higher indicating a problematic level of fatigue [41].

### Statistical analyses

Analyses were conducted using STATA 13.1, except for correction for multiple testing, which was calculated in R 3.3.2. Selective dropout between baseline and the 12-month measurement was tested using the same procedure as in the evaluation of the short-term effectiveness [29]. A logistic regression analysis was conducted with dropout (0 = no, 1 = yes) as outcome and research condition, demographic and disease-related characteristics, and baseline values of the health outcomes (i.e., functional scales of the EORTC QLQ-C30, anxiety and depression scales of the HADS, and total fatigue scale of the CIS) as independent variables.

#### Main outcome analyses

To evaluate the long-term effectiveness of the KNW on emotional functioning, social functioning, depression, and fatigue, we conducted multilevel linear regression analyses (mixed models) with a random intercept for three levels (1: time; 2: individual; 3: hospital), and research condition and the baseline value of the outcome variable as random slopes within the hospital level. The models were kept similar to the models used to evaluate the short-term effectiveness of the KNW [29], except that time was added as an additional level. By including an interaction term between condition (0 = control, 1 = intervention) and time (0 = 6 months, 1 = 12 months) it could be evaluated whether the effects at 6 months from baseline remained at 12 months from baseline (with a non-significant interaction indicating that the intervention effects do not change over time). The condition variable in this model reflects the intervention effectiveness at 6 months from baseline [42]. By recoding the time variable (0 = 12 months, 1 = 6 months), the condition variable in the model reflects intervention effectiveness at 12 months from baseline. Recoding the time variable is a more efficient way to determine the intervention effectiveness at particular time points than conducting simple slope analyses, while providing exactly the same results [42, 43].

Results are provided for the crude models (unadjusted models with only the variables condition, time, and condition*time included in the model) as well as the adjusted models [42]. The adjusted models were corrected for gender, age, relationship status, educational level, income level, employment status, BMI, cancer type, having had cancer before, treatment type, time since last treatment, participation in support program after treatment, comorbidity, and dropout characteristics. Categorical variables with more than two categories were dummy coded. The multilevel models were fit using the maximum likelihood procedure. To correct for multiple testing, we applied the false discovery rate (fdr) method [44]. Cohen’s *d* was provided for insight into the effect sizes of the intervention effects [45]. Intention-to-treat analyses were conducted by imputing data for participants who did not fill in the 6-month or 12-month questionnaire by means of multiple imputation. Missing data was imputed 20 times and based on the same predictors used in the mixed models [46].

Further, we evaluated whether module use influenced the long-term intervention effects using the same procedure as in the evaluation of the short-term effectiveness [29]. A module was considered used when participants continued after visiting the introduction page of the module. With quality of life being considered as a global measure of intervention effectiveness [28], we tested whether the number of modules used influenced the effects on emotional and social functioning. This was done by categorizing the condition variable into three categories: control group, participants who made little use of the KNW (i.e., visited 0–1 modules), and participants who made more intensive use of the KNW (i.e., visited 2–8 modules).^1^ For depression and fatigue, we tested whether the effects differed among participants who visited the modules addressing depression and fatigue, respectively. This was done by recategorizing the condition variable into: control group, module Mood/Fatigue used, module Mood/Fatigue not used. For the analyses on the effects of module use, only the data of baseline and 12-month follow-up was used.

#### Moderator analyses

In order to explore whether the short- and long-term intervention effects differed among specific subgroups of participants, moderator analyses were conducted. In the moderator analyses for the short-term effects, only the 6-month follow-up data was included, and for the long-term effects moderator analyses, only the data of the 12-month follow-up was included. Interaction terms between intervention condition and age, gender, educational level, and treatment type were assessed. To get better insight into the influence of different treatment modalities on the intervention effectiveness, treatment type was recategorized into “surgery alone,” “chemotherapy with or without surgery,” “radiotherapy with or without surgery,” and “chemotherapy and radiotherapy with or without surgery” (see Table 1), with “surgery alone” used as reference category. Since interaction terms have less power, the significance levels of the interaction terms were set to *p* < .10 [42]. When an interaction term was significant, the subgroup effects for gender, educational level (dummy coded), and treatment type (dummy coded) were determined according to the same procedure as the subgroup effects for the interaction between time and condition were determined in the longitudinal mixed models. For example, when entering the interaction between condition (0 = control, 1 = intervention) and gender (0 = male, 1 = female), the coefficient for condition indicates the intervention effectiveness for men. By recoding the gender variable, the coefficient for condition indicates the intervention effectiveness for women. Age was entered as a continuous variable. When the interaction between age and condition was significant, margins were plotted to determine the cutoff point for which age group the intervention was effective [47]. Then, age was dichotomized and the effectiveness for the different age groups was determined using the same procedure as the other binary moderators.

## Results

**Fig. 1:**
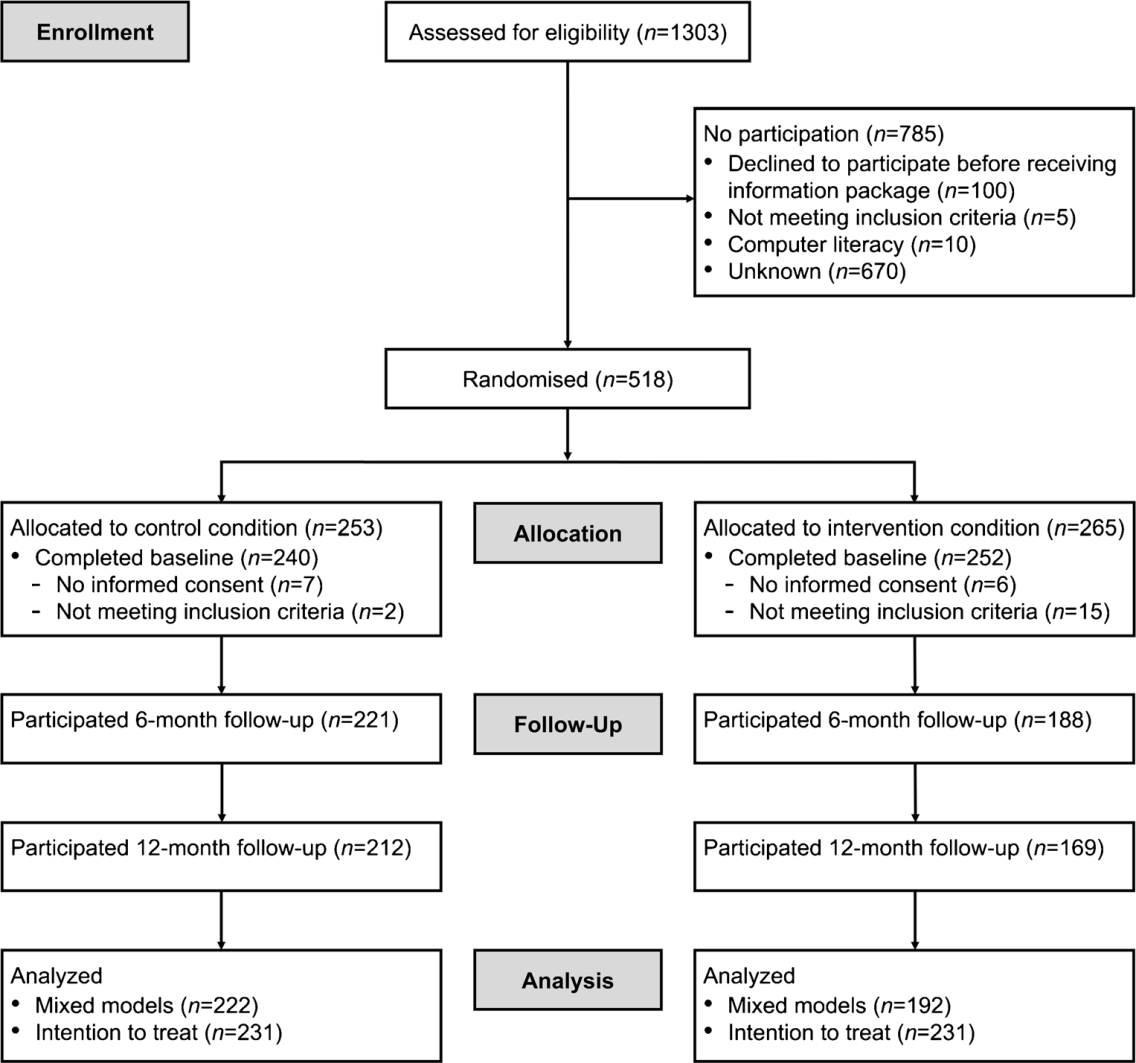
Flow diagram of the study

An overview of the number of patients enrolled in the intervention and lost to follow-up is provided in Fig. 1. Patient characteristics are displayed in Table 1. Dropout analyses showed that participants in the control group (*B* = 1.73, *SE* = 0.32, *p* < .001) and participants with approximately modal income (in comparison to below modal income) (*B* = −0.82, *SE* = 0.41, *p* = .046) were more likely to fill in the 12-month questionnaire, while participants with higher social functioning were less likely to do so (*B* = 0.02, *SE* = 0.01, *p* = .032) Of the overall sample at baseline, 13.4% had a clinical indication for depression and 34.9% for fatigue according to the manual instructed cutoff scores.

### Intervention use and appreciation

The participants in the intervention group who completed the 6- or 12-month measurement (*n* = 192) on average used 2.20 modules (*SD* = 1.58). Of those using at least one module (89.1%), the average time between first login and last use of a module was 10.63 weeks (*SD* = 6.78), with 84.8% using a module for the last time within 18 weeks since first login. In total, 30.2% used one module, 18.8% used two modules, 21.9% used three modules, 9.9% used four modules, and 8.3% used five or more modules. Visitor rate per module is Diet 60.9%, Fatigue 37.0%, Return to work 24.5%, Mood (anxiety and depression) 24.0%, Physical activity 24.0%, Residual symptoms 20.8%, Relationships 18.8%, and Smoking 9.9%. The overall appreciation of the KNW, on a scale from 1 to 10, was considered high (*M* = 7.48, *SD* = 1.20) [34].

### Intervention effects

**Fig. 2:**
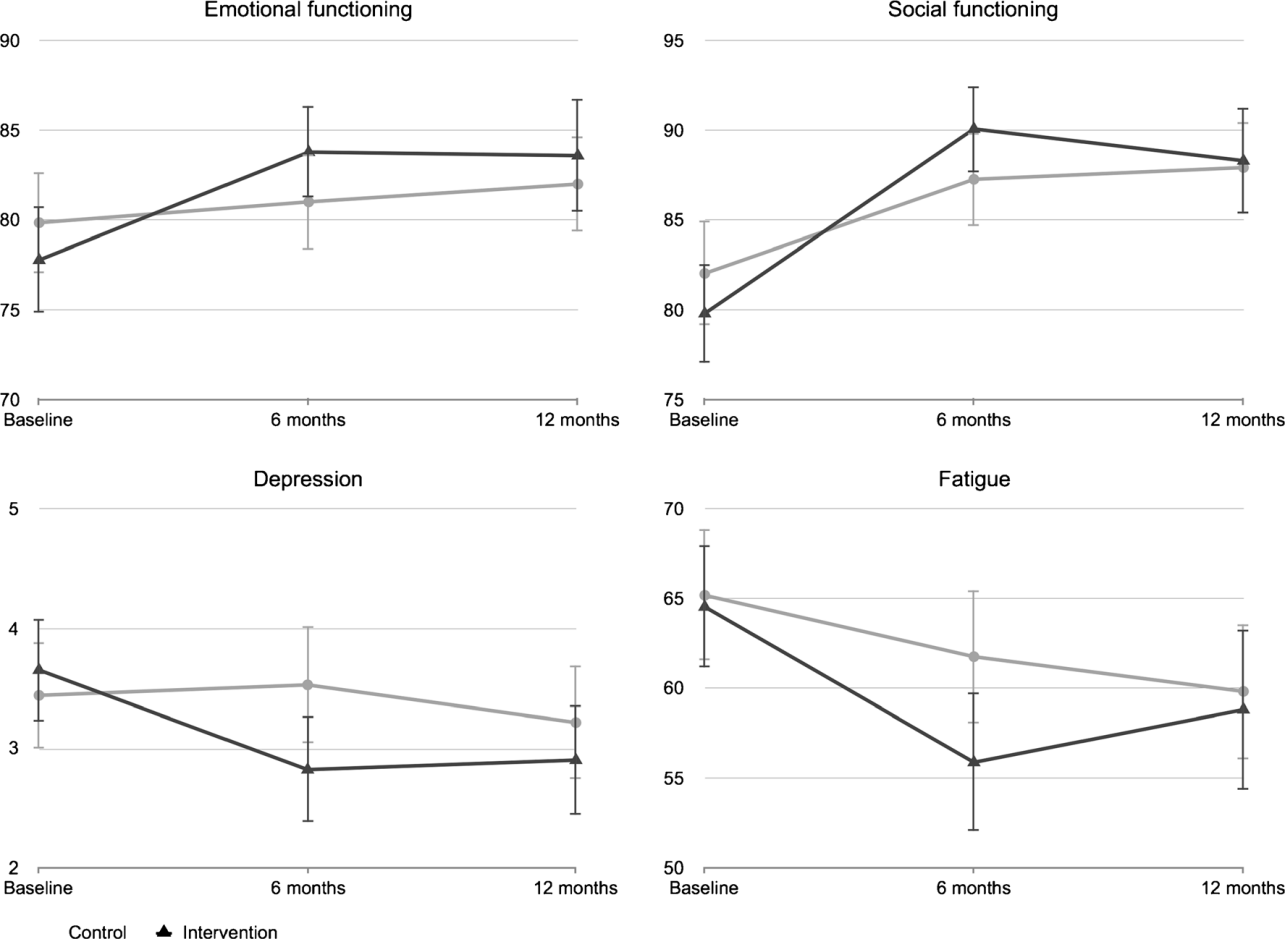
Line graphs of the outcome scores at baseline and 6 and 12 months after baseline. *Vertical bars* represent the 95% CI of the mean

Table 2 and Fig. 2 show the mean scores of the outcomes at baseline and at 6 and 12 months after baseline (see Online Resource 2 for a more detailed overview of the data distribution). The interaction terms between time and condition for emotional functioning (*B* = −0.39, *SE* = 1.66, *p* = .814), social functioning (*B* = −2.20, *SE* = 2.02, *p* = .276), depression (*B* = 0.21, *SE* = 0.24, *p* = .376), and fatigue (*B* = 3.03, *SE* = 1.93, *p* = .117) were non-significant, indicating that the intervention effects remain over time. However, the directions of the interaction coefficients indicate that the differences in the outcomes between the intervention and control group are smaller at 12 months from baseline than they were at 6 months from baseline. As a result, between-group differences at 12 months from baseline on emotional (*B* = 2.65, *SE* = 1.60, *p* = .096, *d* = 0.08) and social functioning (*B* = 1.31, *SE* = 1.67, *p* = .435, *d* = 0.02), depression (*B* = −0.25, *SE* = 0.21, *p* = .227, *d* = 0.10), and fatigue (*B* = −1.01, *SE* = 1.98, *p* = .611, *d* = 0.04) were all non-significant (Table 3). The mean scores on the outcomes suggest that the intervention group remained fairly stable in emotional and social functioning, depression and fatigue between 6 and 12 months from baseline, but that the control group slightly improved over time, leading to non-significant group differences at 12 months from baseline. Furthermore, no effects were found for the influence of module use on the 12-month intervention effects.

**Table 2:** Means and *SD*’s of outcomes at baseline and 6 and 12 months

	Baseline (*n* = 462)	6 months (*n* = 409)	12 months (*n* = 379)
Emotional functioning			
Control	79.83 ± 21.49	81.00 ± 20.31	81.90 ± 19.61
Intervention	77.78 ± 22.60	83.78 ± 17.76	83.58 ± 20.58
Social functioning			
Control	82.03 ± 22.53	87.25 ± 19.45	87.86 ± 19.00
Intervention	79.80 ± 21.04	90.07 ± 16.86	88.29 ± 19.52
Depression			
Control	3.44 ± 3.45	3.53 ± 3.67	3.21 ± 3.47
Intervention	3.65 ± 3.26	2.82 ± 3.06	2.90 ± 2.99
Fatigue			
Control	65.20 ± 28.25	61.77 ± 28.15	59.87 ± 27.51
Intervention	64.55 ± 26.46	55.90 ± 26.72	58.83 ± 29.14

**Table 3:** Results of the multilevel analyses testing the effectiveness of the KNW on emotional and social functioning, depression, and fatigue at 6 and 12 months from baseline

		Mixed models (*n* = 414)						Imputed data (*n* = 462)			
		*B*	SE *B*	95% CI	*p*	*p*_*fdr*_	*d* [95% CI]	*B*	SE *B*	95% CI	*p*
Emotional functioning											
6 months	Crude	3.21	1.55	0.18–6.25	.038	.038	−0.15 [−0.34–0.05]	2.35	1.59	−0.77–5.48	.139
	Adjusted	3.04	1.54	0.02–6.07	.049	.049		1.92	1.57	−1.15–5.00	.221
12 months	Crude	2.79	1.60	−0.35–5.93	.081	.661	−0.08 [−0.28–0.12]	0.27	1.67	−3.01–3.56	.871
	Adjusted	2.65	1.60	−0.47–5.78	.096	.384		−0.16	1.66	−3.43–3.11	.923
Social functioning											
6 months	Crude	3.64	1.65	0.41–6.87	.027	.037	−0.15 [−0.35–0.04]	1.37	1.83	−2.22–4.96	.453
	Adjusted	3.50	1.61	0.35–6.66	.030	.048		1.03	1.78	−2.45–4.53	.562
12 months	Crude	1.38	1.71	−1.97–4.73	.421	.661	−0.02 [−0.22–0.18]	−3.01	1.81	−6.57–0.54	.096
	Adjusted	1.31	1.67	−1.97 – 4.59	.435	.580		−3.36	1.76	−6.80–0.10	.057
Depression											
6 months	Crude	−0.51	0.20	−0.90–−0.11	.011	.037	0.21 [0.01–0.40]	−0.51	0.21	−0.93–−0.10	.014
	Adjusted	−0.46	0.20	−0.86–−0.07	.021	.048		−0.41	0.21	−0.82–−0.00	.049
12 months	Crude	−0.30	0.21	−0.70–0.10	.145	.661	0.10 [−0.11–0.30]	−0.19	0.21	−0.60–0.23	.375
	Adjusted	−0.25	0.21	−0.66 – 0.16	.227	.454		−0.09	0.21	−0.50–0.33	.684
Fatigue											
6 months	Crude	−4.36	1.98	−8.23–−0.48	.028	.037	0.21 [0.02–0.41]	−4.84	1.96	−8.68–−1.00	.014
	Adjusted	−4.04	1.92	−7.82–−0.26	.036	.048		−4.12	1.91	−7.87–−0.39	.030
12 months	Crude	−1.42	2.03	−5.41–2.56	.482	.661	0.04 [−0.17–0.24]	−0.71	2.02	−4.68–3.26	.725
	Adjusted	−1.01	1.98	−4.90–2.88	.611	.661		−0.00	1.98	−3.88–3.88	1.000

*p*_*fdr*_ gives the *p*-values corrected for multiple testing using false discovery rate [44]. Correction for multiple testing was carried out in four sets: (1) crude analyses at 6 months, (2) adjusted analyses at 6 months, (3) crude analyses at 12 months, and (4) adjusted analyses at 12 months

### Moderators

Gender moderated the 6-month effectiveness of social functioning (*p* = .098), with the KNW being effective in improving social functioning for men (*B* = 9.14, *SE* = 3.51, *p* = .009, *d* = 0.34), but not for women (*B* = 2.65, *SE* = 1.74, *p* = .129, *d* = 0.11). Age moderated the 6-month effect of fatigue (*p* = .036), with the KNW being effective in decreasing fatigue for participants aged 56 or younger (*B* = −10.48, *SE* = 2.63, *p* < .000, *d* = 0.44), but not for participants aged 57 or older (*B* = 1.86, *SE* = 2.64, *p* = .480, *d* = 0.02) (see Online Resource 3 Fig. 1). There was also an indication that age moderated the 12-month effect of social functioning (*p* = .098). However, the margin plot showed no significant difference at different values of age (see Online Resource 3 Fig. 2). Educational level moderated the 12-month effect of social functioning (*p*_medium_ = .004, *p*_high_ = .053).^2^ Participants with low educational level reported lower social functioning (*B* = −5.84, *SE* = 2.89, *p* = .043, *d* = .22) and participants with medium educational level reported higher social functioning (*B* = 6.13, *SE* = 2.91, *p* = .035, *d* = .19) than participants with a similar educational level in the control group, while there was no effect for participants with a high educational level (*B* = 2.22, *SE* = 3.02, *p* = .464, *d* = .05). While there was an indication that educational level moderated the 12-month effect of fatigue (*p*_medium_ = .057), no subgroup effects were found. Finally, treatment type moderated the 6-month effect of depression (*p*_cs_ = .027, *p*_crs_ = .083),^3^ with the KNW being effective in decreasing depression for participants who received chemotherapy with or without surgery (*B* = −1.16, *SE* = 0.43, *p* = .008, *d* = 0.36), but not for participants who received surgery only (*B* = 0.58, *SE* = 0.65, *p* = .370, *d* = 0.16), radiotherapy with or without surgery (*B* = −0.47, *SE* = 0.55, *p* = .389, *d* = 0.15), or chemotherapy and radiotherapy with or without surgery (*B* = −0.71, *SE* = 0.36, *p* = .052, *d* = 0.13). For an overview of the means and *SD*s of the health outcomes by moderators, see Online Resource 4.

## Discussion

The current study investigated whether the short-term effects (i.e., 6 months from baseline) of the web-based computer-tailored KNW intervention on emotional and social functioning, depression, and fatigue remained in the long term (i.e., 12 months from baseline). In addition, subgroup differences in intervention effectiveness in the short term and long term were explored. The significant increase on emotional and social functioning and decrease in depression and fatigue at 6 months from baseline within the intervention group (see also [29]) remained fairly stable between 6 and 12 months after baseline, which can be considered as a positive outcome. The control group illustrated a different course, with well-being slowly increasing over the trial period, leading to non-significant differences between the intervention and control group in the long term. Intention-to-treat analyses supported these results. Thus, while the health outcomes of the intervention and control group in the long term are similar, the results suggest that the intervention group benefitted from an extra, earlier period of improved quality of life.

Other eHealth applications for cancer survivors also found that the additional health effects of eHealth interventions did not remain significant in comparison to the control condition after the trial period had ended [26, 48, 49]. In a comparable intervention by van den Berg et al. [26], who evaluated the effectiveness of a non-tailored web-based CBT-based intervention aimed at reducing distress and improving empowerment among breast cancer survivors, the intervention was found to be effective in reducing psychological distress directly after intervention closure, but the intervention and control group did not differ at long-term follow-up. Likewise, the natural recovery of the control group led to non-significant between-group differences at long-term follow-up. For face-to-face interventions, long-term effects, albeit often reduced in part, more frequently sustain [50, 51]. This suggests that face-to-face interventions seem to be more effective in sustaining effects in the long term than eHealth intervention do. However, since there is little evidence on the long-term effects of eHealth interventions for cancer survivors, more research is needed to verify this idea.

The KNW was developed for the general population of cancer survivors as a tool to provide personalized information on what to expect after cancer treatment and how to effectively address the survivors’ most prominent needs and problems. As the KNW was available for all cancer survivors, there was no pre-selection on baseline values of the outcomes quality of life, depression, and fatigue. As a consequence, a minority of the participants experienced strong problems on these issues. The fact that, in the long term, no further improvement in emotional and social functioning and no further decrease in depression occurred in the intervention group might therefore be explained by the study sample scoring fairly well on these outcomes. The 6-month scores were already quite good and hard to further improve, and maintenance of these scores should be considered as being very positive. It could be expected that the intervention might be more effective for participants scoring less well on baseline quality of life and depression, since they would have greater opportunity for improvement than those who functioned better at the start of the intervention [31, 50, 52]. Since there was little variation in the baseline score of these health outcomes within the current study, moderation analyses would provide unreliable results. Therefore, future research should investigate how the effects of the KNW differ between groups with more or less experienced problems.

For fatigue, further improvement would have been possible, but the intervention group showed no further decrease in fatigue after the 6-month measurement. The restricted time period that the intervention was available for use might be a possible explanation for this effect [26]. Fatigue is a multidimensional and complex problem, which can be treated from different angles, such as CBT for fatigue, depression, or sleep; psychoeducation; regulating activities; physical exercise; and relaxation exercises [6, 53]. While most of these aspects were discussed within the KNW, the amount of information and assignments might be too extensive for this restricted time period. On the other hand, user statistics of the KNW showed that the majority of the participants (>80%) stopped using any of the modules after 18 weeks following first use [29]. This long-term discontinuation of eHealth application usage is a widely recognized phenomenon [54, 55]. It is therefore suggested that new techniques can be added to the intervention that may improve the provided feedback and advices and facilitate participant engagement in the intervention. They subsequently need to be tested for their added value. One suggestion could be to add face-to-face support to the intervention, changing the intervention into a blended approach [51, 55, 56]. However, the downside of adding face-to-face support is that, because of therapist burden, it could undermine the high reach of the intervention [55], while costs would increase significantly. Another option is to provide more additional long-term tailored feedback on the participants’ change of their behavior and health status over time [57]. While the KNW provided direct feedback after assignment completion and several e-mail reminders to complete the module were sent, no updates were given on the progress over time in comparison to the baseline outcome. By providing such feedback on the survivors’ well-being and behavioral changes at multiple moments, long-term effectiveness can be enhanced [57].

Nonetheless, the results are highly relevant, as they suggest that the KNW expedites recovery after cancer. In practice, this means that an easily accessible, low intensity, and fairly inexpensive intervention can offer several extra months of increased quality of life for a large group of early cancer survivors. This might imply that the health benefits gained from the KNW may lead to lower psychological comorbidity or mortality [58–60], which might result in better adherence to follow-up treatment [61], or better or earlier integration in daily life (e.g., social relations, return to work) [62, 63]. Therefore, the KNW can serve as a relevant step in a stepped care approach in cancer aftercare [17, 18, 64]. For patients with mild problems who are able to manage these problems themselves with personalized information and guidance, the KNW can offer sufficient and adequate support to increase and accelerate their recovery process. For patients with more severe problems who might need more intensive support (e.g., face-to-face therapy, or medication), the KNW can recommend seeking out this additional support.

### Moderators

The results of the moderator analyses indicate that the KNW was more effective for specific subgroups. In the short term, the KNW was primarily effective in improving social functioning for men (*d* = 0.34), decreasing fatigue for those aged 56 and younger (*d* = 0.44), and decreasing depression for those who received chemotherapy with or without surgery (*d* = 0.36). The effect sizes for these subgroups were substantially higher in comparison to the effect sizes of the complete sample. In the long term, participants with a medium educational level reported higher social functioning (*d* = 0.19), while participants with a low educational level reported lower social functioning (*d* = 0.22) than participants with a similar educational level in the control group. These effect sizes were relatively small.

Concerning the moderating effect of age on fatigue, another study exploring moderators of a guided self-instruction intervention for chronic fatigue syndrome also found the intervention to be more effective for younger patients [65]. A possible explanation could be that the prognosis for older patients experiencing fatigue is worse than for younger patients, and therefore the KNW could not offer sufficient support for the older population. While some studies support this relation between older age and worse prognosis, this evidence is mixed [60]. Another explanation could be that younger patients might be more pro-active to address their experienced problems, while older patients tend to be more accepting of their physical decline [66].

The finding that the KNW was more effective for participants who received chemotherapy might be explained by the finding that patients who have received chemotherapy might be more at risk of developing depressive symptoms [67, 68]. It is possible that the sections within the KNW that discussed depressive feelings addressed the needs of this subgroup better. It should be noted that, while almost significant, an intervention effect for depression was not found for the participants who received both chemotherapy and radiotherapy with or without surgery. Possibly, a light intervention as KNW might not be powerful enough to effectively address stronger feelings of depression in some of those who received the heaviest and most intrusive cancer treatment.

Concerning social functioning, results suggest that, in the short term, the intervention had a greater effect on men than women in feeling that their physical condition or treatment did not interfere with their family life or social activities. One explanation could be that women in general rely on a broader social support network of family, friends, and partner in dealing with cancer-related distress, while men primarily find support in their partner [69]. Possibly, the advices provided within the KNW on dealing with social relationships focus less on support in dealing with more complex social relationship structures.

The differences in effect on social functioning within educational level in the long term were unexpected, since the moderator analyses at the short term showed no difference in direction of intervention effects. Investigation of the mean scores of social functioning by educational level (Online Resource 4) suggest that people with a low educational level in the control group show a “natural” recovery over time. The participants in the intervention group with a low educational level also increase in social functioning 6 months from baseline, but show a small drop at 12 months from baseline. Thus, it is not the case that participants with a low educational level in the intervention group report worse social functioning in the long term in comparison to baseline. Instead, the control group improves more in social functioning than the intervention group does. Therefore, the intervention is not considered effective in increasing social functioning for participants with a low educational level. For participants with a medium educational level, the mean scores suggest that the intervention group improved in social functioning at 6 months from baseline and this effect remained at the long term. Therefore, the intervention is considered effective in improving social functioning for participants with a medium educational level.

The moderator analyses were exploratory and therefore the results need to be interpreted with caution. They do, however, provide useful insight in directions in which the intervention and the tailored advices within the intervention could be improved. For example, further development of the intervention could focus on how to address the needs of elderly users better or how to adjust the advices so they match better to the issues associated with the received treatment type.

### Limitations

There are some limitations that should be mentioned. First, selective dropout might have influenced the results, in particular the higher dropout in the intervention group. This differential dropout is not uncommon in health behavioral change trials and might be explained by the intervention being too time intensive or the intervention not meeting the participant’s expectations [70]. Fortunately, the dropout rate at 6 and 12 months from baseline was very low (11.5 and 17.5%, respectively). With this low dropout rate and correction for the differences between completers and non-completers in the analyses, minimal influences of dropout effects may be expected. In addition, the intention-to-treat analyses supported the findings of the long-term effectiveness. Second, while the study aimed to recruit a diverse group of cancer survivors, women with breast cancer and survivors who scored fairly well on quality of life and depression were over-represented. Furthermore, the intervention reached the older population to a lesser extent. In general, female, younger, and low-risk individuals are more prone to participate in online interventions [55, 71]. Nonetheless, this selection bias makes it more difficult to generalize the finding to the general population of cancer survivors. Third, while the sensitivity to change for both the CIS and the EORTC QLQ-C30 seems to be adequate [72–74], evidence on the sensitivity to change for the HADS is mixed [75, 76]. Thus, the finding that there were no long-term effects on depression could be attributable to the scale not being responsive enough. However, because of little evidence, no strong conclusions can be made on this point. Finally, the online questionnaires were self-administered. While only validated questionnaires were used, the results could be influenced by social desirability.

## Conclusion

The results of the current study add new insights to the scarce evidence of the (long-term) effectiveness of eHealth interventions for cancer survivors [19]. These results support the notion that web-based interventions can speed up the recovery process of cancer survivors. It is expected that the KNW in particular will be effective for survivors without medical indication, who are experiencing milder complaints that they can manage themselves with the right personalized information [18]. With the KNW being a relatively low intensity, easily accessible, and low cost tool, which has the potential to reach a large group of cancer survivors, it is believed that it could adequately serve as a relevant step in stepped care for the larger population of cancer survivors, helping them to more quickly deal with their experienced problems and therefore accelerate their recovery.

## Electronic supplementary material

Online Resource 1 (PDF 938 kb).

Online Resource 2 (PDF 399 kb).

Online Resource 3 (PDF 433 kb).

Online Resource 4 (PDF 404 kb).

## Footnotes

1 Since a small percentage of participants used four or more modules (see the “Results” section), number of modules used was not suitable for use as a continuous variable and, therefore, was dichotomized using a median split.

2
*p*_*medium*_ indicates the significance level of the interaction term between condition and medium educational level. *p*_*high*_ indicates the significance level of the interaction term between condition and high educational level.

3
*p*_*cs*_ indicates the significance level of the interaction term between condition and the treatment type category “chemotherapy with or without surgery.” *p*_*crs*_ indicates the significance level of the interaction term between condition and the treatment type category “chemotherapy and radiotherapy with or without surgery.”
