# Supplementary material for: Long-term effectiveness and moderators of a web-based tailored intervention for cancer survivors on social and emotional functioning, depression, and fatigue: randomized controlled trial
Source: J Cancer Surviv. 2017 Jul 11;11(6):691–703. doi: 10.1007/s11764-017-0625-0 (PMC5671537; doi:10.1007/s11764-017-0625-0)
Supplement: Supplementary file 1 — (PDF 938 kb). [file 11764_2017_625_MOESM1_ESM.pdf]

# ONLINE RESOURCE 1

*Article title:* Long-term effectiveness and moderators of a web-based tailored intervention for cancer survivors on social and emotional functioning, depression, and fatigue: randomized controlled trial
*Journal:* Journal of Cancer Survivorship
*Authors:* Roy A. Willems, Ilse Mesters, Lilian Lechner, Iris M. Kanera, Catherine A.W. Bolman
*Contact:* Roy Willems, Faculty of Psychology and Educational Sciences, Open University of the Netherlands, P.O. Box 2960, 6401DL, Heerlen, The Netherlands
*E-mail:* roy.willems@ou.nl

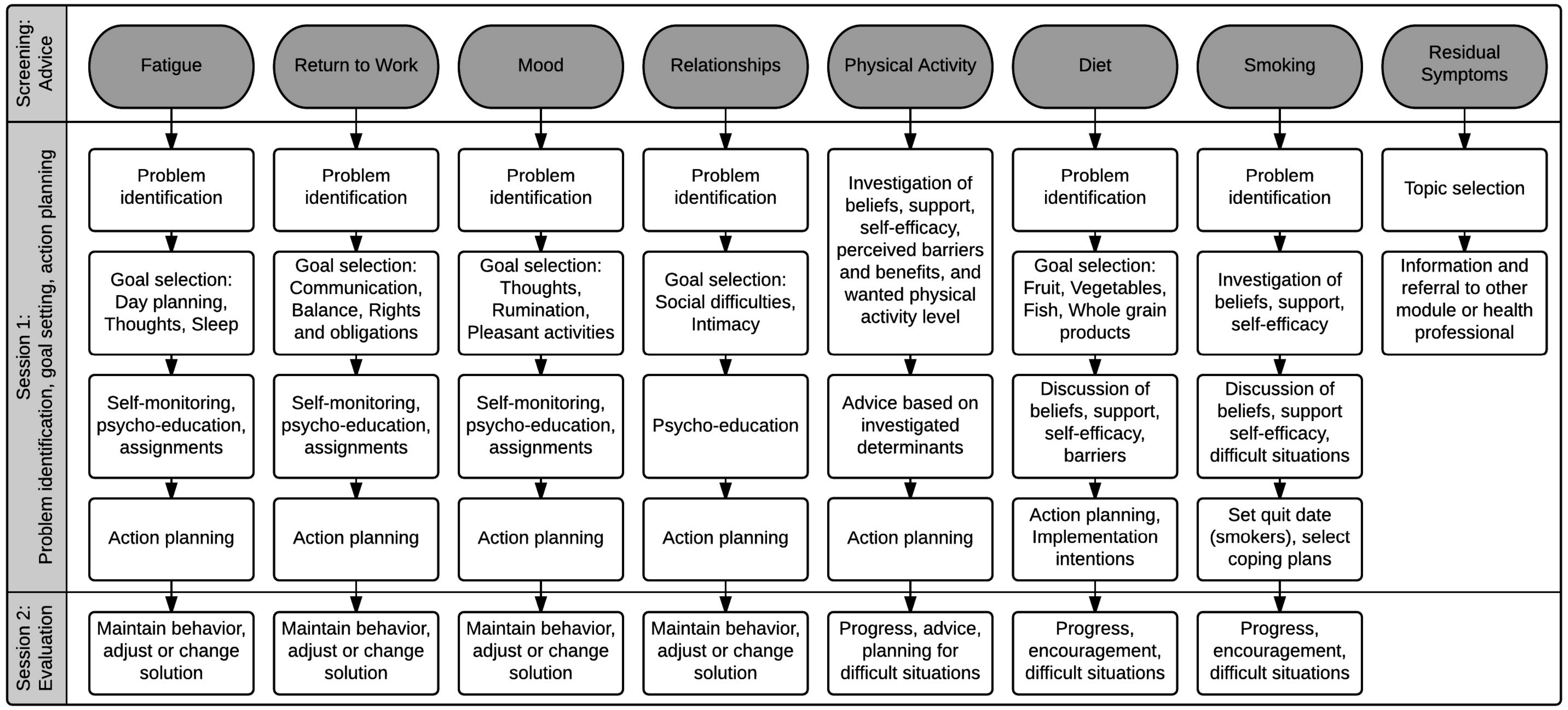


Overview of the scope and sequence of the modules. From Willems et al. (2015). © 2015 Willems et al. Reprinted with permission.
