# Supplementary material for: Long-term effectiveness and moderators of a web-based tailored intervention for cancer survivors on social and emotional functioning, depression, and fatigue: randomized controlled trial
Source: J Cancer Surviv. 2017 Jul 11;11(6):691–703. doi: 10.1007/s11764-017-0625-0 (PMC5671537; doi:10.1007/s11764-017-0625-0)
Supplement: Supplementary file 3 — (PDF 433 kb). [file 11764_2017_625_MOESM3_ESM.pdf]

ONLINE RESOURCE 3

Article title: Long-term effectiveness and moderators of a web-based tailored intervention for cancer survivors on social and emotional functioning, depression, and fatigue: randomized controlled trial
Journal: Journal of Cancer Survivorship
Authors: Roy A. Willems, Ilse Mesters, Lilian Lechner, Iris M. Kanera, Catherine A.W. Bolman
Contact: Roy Willems, Faculty of Psychology and Educational Sciences, Open University of the Netherlands, P.O. Box 2960, 6401DL, Heerlen, The Netherlands
E-mail: roy.willems@ou.nl

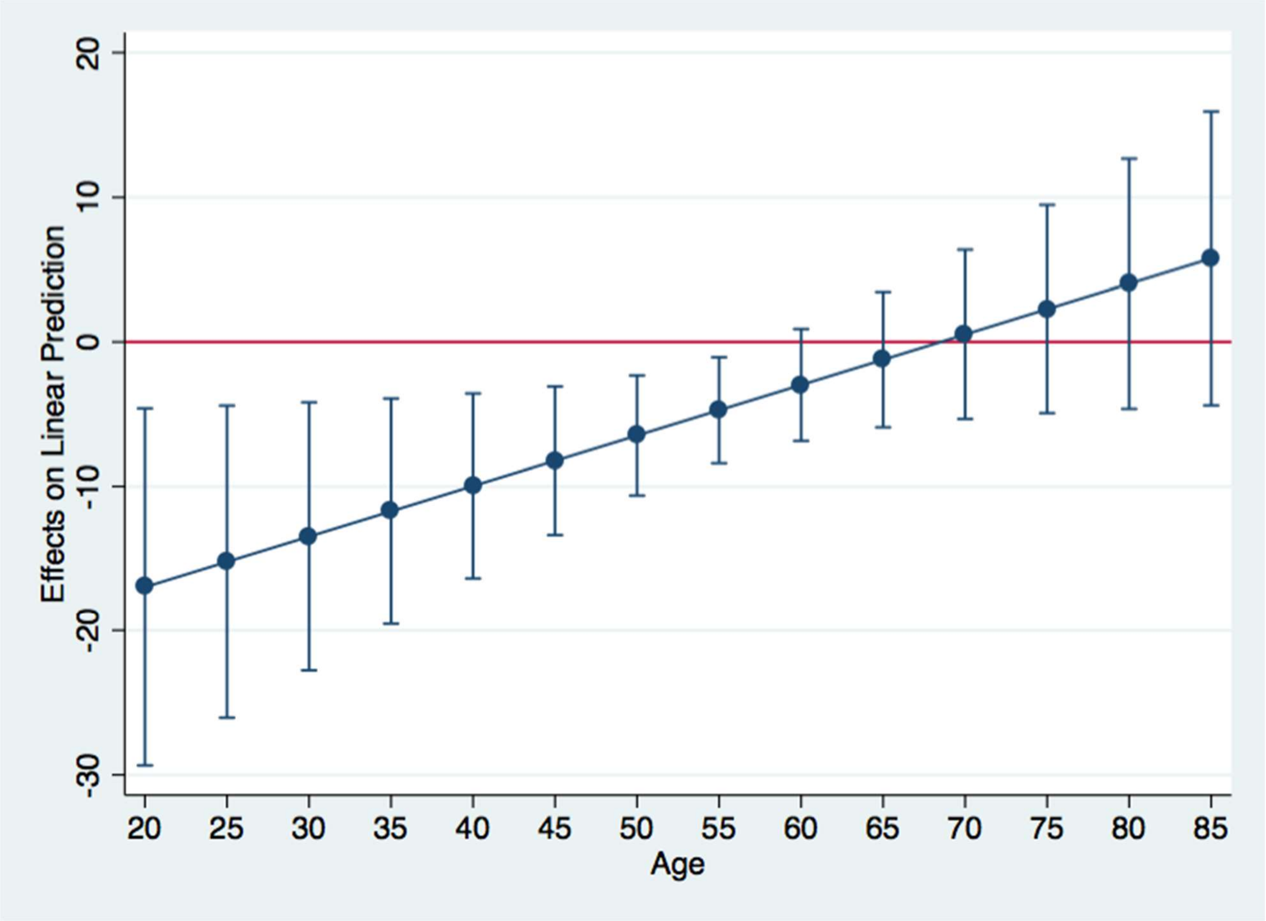

Figure 1. Average marginal effects of condition on fatigue at 6 months from baseline for different ages. Vertical bars represent the 95% CI.

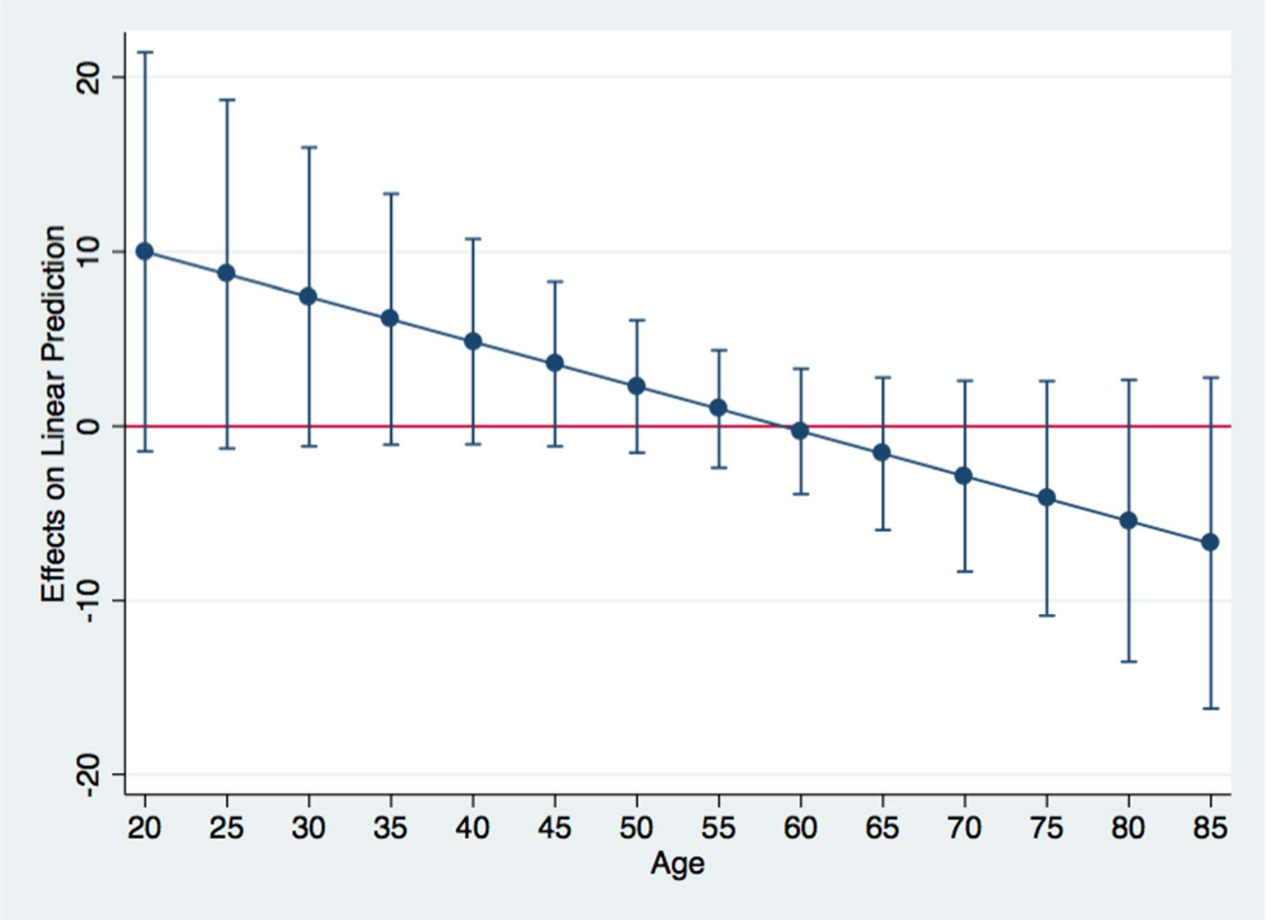

Figure 2. Average marginal effects of condition on social functioning at 12 months from baseline for different ages. Vertical bars represent the 95% CI.
