# Supplementary material for: Long-term effectiveness and moderators of a web-based tailored intervention for cancer survivors on social and emotional functioning, depression, and fatigue: randomized controlled trial
Source: J Cancer Surviv. 2017 Jul 11;11(6):691–703. doi: 10.1007/s11764-017-0625-0 (PMC5671537; doi:10.1007/s11764-017-0625-0)
Supplement: Supplementary file 4 — (PDF 404 kb). [file 11764_2017_625_MOESM4_ESM.pdf]

ONLINE RESOURCE 4

Article title: Long-term effectiveness and moderators of a web-based tailored intervention for cancer survivors on social and emotional functioning, depression, and fatigue: randomized controlled trial
Journal: Journal of Cancer Survivorship
Authors: Roy A. Willems, Ilse Mesters, Lilian Lechner, Iris M. Kanera, Catherine A.W. Bolman
Contact: Roy Willems, Faculty of Psychology and Educational Sciences, Open University of the Netherlands, P.O. Box 2960, 6401DL, Heerlen, The Netherlands
E-mail: roy.willems@ou.nl

Means and SDs for fatigue, depression, and social functioning by moderators

		Control		Intervention	
		n	mean ± *SD*	*n*	mean ± *SD*
Fatigue by age					
≤ 56 years	T0	107	69.86 ± 27.33	125	68.38 ± 25.39
	T2	101	66.40 ± 29.45	97	54.43 ± 25.17
	T3	96	62.53 ± 27.82	88	60.38 ± 30.11
≥ 57 years	T0	124	61.18 ± 28.52	106	60.05 ± 27.10
	T2	120	57.88 ± 26.52	91	57.47 ± 28.34
	T3	115	57.57 ± 27.06	80	57.14 ± 28.12
Depression by treatment type					
Surgery only	T0	31	4.32 ± 4.04	28	3.14 ± 3.08
	T2	29	3.90 ± 3.76	25	3.32 ± 3.66
	T3	26	3.54 ± 4.15	21	3.19 ± 3.74
Chemotherapy ± surgery	T0	58	3.43 ± 3.19	69	3.52 ± 2.95
	T2	58	3.81 ± 3.94	53	2.55 ± 3.03
	T3	54	3.43 ± 3.73	44	2.84 ± 2.84
Radiotherapy ± surgery	T0	33	3.91 ± 3.67	47	3.91 ± 3.63
	T2	31	3.19 ± 3.43	43	2.67 ± 3.31
	T3	30	3.20 ± 3.31	39	2.21 ± 2.69
Chemotherapy and radiotherapy ± surgery	T0	109	3.06 ± 3.32	87	3.78 ± 3.37
	T2	103	3.37 ± 3.61	67	2.96 ± 2.71
	T3	101	3.03 ± 3.21	64	3.28 ± 2.99
Social functioning by gender					
Men	T0	45	85.19 ± 21.97	48	81.94 ± 18.78
	T2	42	88.10 ± 21.87	36	94.44 ± 14.36
	T3	41	90.24 ± 19.36	29	90.80 ± 18.68
Women	T0	186	81.27 ± 22.67	183	79.23 ± 21.61
	T2	179	87.06 ± 18.91	152	89.04 ± 17.27
	T3	170	81.35 ± 18.89	139	87.77 ± 19.72
Social functioning by educational level					
Low	T0	97	77.66 ± 24.87	76	78.95 ± 19.12
	T2	93	86.38 ± 19.50	63	88.36 ± 17.87
	T3	87	87.55 ± 18.19	52	83.33 ± 20.87
Medium	T0	70	86.19 ± 18.16	76	80.26 ± 20.85
	T2	65	84.36 ± 20.60	64	89.58 ± 17.44
	T3	61	85.79 ± 21.48	62	89.78 ± 20.99
High	T0	64	84.11 ± 22.31	79	80.17 ± 23.12
	T2	63	91.53 ± 17.68	61	92.35 ± 15.08
	T3	63	90.48 ± 17.38	54	91.36 ± 15.44

T0 = baseline, T2 = 6 months from baseline, T3 = 12 months from baseline
